# Cardiovascular risk in children: a burden for future generations

**DOI:** 10.1186/s13052-022-01250-5

**Published:** 2022-04-11

**Authors:** Maria Candelino, Veronica Maria Tagi, Francesco Chiarelli

**Affiliations:** Department of Paediatrics, University of Chieti, Chieti, Italy

**Keywords:** Congenital heart diseases, Congenital coronary anomalies, Kawasaki disease, Chronic kidney disease, Childhood cancer, Children

## Abstract

Cardiovascular diseases are the main causes of death and health costs in developed countries. Although cardiovascular diseases are thought to affect only adulthood, the underlying process of atherosclerosis begins in the first decade of life. Epidemiological studies show that severity of atherosclerosis depends both on the number and intensity of risk factors. Early detection of cardiovascular risk in childhood is the most powerful tool to prevent cardiovascular accidents in adulthood and possibly reduce its consequent burden for the future.

A large amount of cardiovascular risk factors is already detectable in childhood and include non-modifiable elements, among which genetic factors and congenital heart diseases, and modifiable elements, which depend on environmental effects (e.g. lifestyle and nutrition). Regardless of the possibility to intervene on these factors, an early diagnosis is fundamental to ensure an optimal life expectancy in adulthood. The most important cardiovascular risk factors in the paediatric age and adolescence are excess weight, arterial hypertension, glucose metabolism and lipid metabolism alterations.

In this review we will discuss the main risk factors strictly correlated with cardiac and vessels diseases, focusing on their pathogenesis, diagnosis, and treatments.

## Introduction

Cardiovascular diseases (CVD) represent the main cause of death and health costs in industrialized countries. Several studies show that early atherosclerosis can be detected in childhood and its progression depends on exposure to several risk factors [1]. In fact, the initial vascular alterations of atherosclerotic pathology appear in the first decade of life [2]. Risk factors include metabolic alterations, hypertension, and chronic inflammatory conditions. Anyway, underlying structural or functional heart diseases are also important causes of CVD in childhood [1].

Some studies, such as the Bogalusa Heart Study and the Muscatine Study, have shown the association between exposure to various conventional cardiovascular risk factors in childhood and the development of subclinical atherosclerosis in old age. In particular, an increase in cholesterol and apolipoprotein levels in childhood is associated with an increase in carotid intima-media thickness (cIMT), a reduction in carotid elasticity and a compromised brachial endothelial function in adulthood [3].

A recent meta-analysis has recommended to perform a screening in children with cardiovascular risk factors from the end of the first decade of life, in order to detect subclinical atherosclerosis, because in children aged 9 years or older an increase in cIMT was predictive of the development of atherosclerosis in adulthood [4].

Besides the origins of atherosclerosis in early childhood, observational and epidemiological studies have shown the direct correlation between the severity and extent of atherosclerosis with the number of risk factors to which the child is exposed during childhood, their intensity, and their combined effect [5]. Furthermore, it has been demonstrated that also low fruit and vegetable consumption and poor physical activity are associated with a rapid progression of arterial inflammation and an increase in cIMT during adulthood [3].

For the aforementioned reasons, paediatricians should consider atherosclerosis as a widespread disease in childhood and therefore adopt preventive strategies since early life both in general population and, in a targeted way, in subjects with increased cardiovascular risk [6].

## Dyslipidaemia

Several studies, such as the Bogalusa Heart Study and the Pathobiological Determinants of Atherosclerosis in Youth Study, have shown that atherosclerosis begins during childhood, in the form of lesions (named fatty streaks), characterized by the presence of lipid-filled macrophages inside the intima of the artery, which are the precursors of atheromatous plaque [7, 8].

Among the various cardiovascular risk factors, LDL cholesterol (LDL-C) is probably the most implicated in the development of atherosclerotic plaques. Continuous exposure to high serum LDL-C levels during adolescence can lead to the accumulation of calcium in the coronary arteries and, consequently, to the development of atherosclerotic plaques in adulthood. This can result in irreversible changes in the coronary arteries and an increased risk of ischemic heart disease in adulthood. Thereafter, aiming to low serum LDL-C levels since childhood is fundamental for the prevention of cardiovascular accidents [9].

Hypercholesterolemia is the most relevant cardiovascular risk factor linked to the progression of coronary heart disease. Its most frequent causes are genetic, being familial hypercholesterolemia (FH) the most relevant [10].

FH is an autosomal dominant genetic disorder of cholesterol metabolism. The heterozygous form affects 1 in 250 individuals and is characterized by very high levels of LDL-C. The diagnosis can be suspected in the presence of LDL-C levels ≥ 160 mg/ dL (4.0 mmol / L) associated with a family history of elevated LDL-C or premature CVD in first or second-degree relatives. The suspect can be confirmed by a positive genetic test for a genetic defect that increases LDL-C [1].

In addition, dyslipidaemias in children can lead to impaired heart function over time, up to heart failure. Hence, early diagnosis of left ventricular hypertrophy (LVH) and of reduced cardiac function is essential in children with dyslipidaemias, to start an appropriate therapy. Screening is important not only for children at risk, but for the entire paediatric population during growth. The non-invasive methods that should be used for risk stratification in children with dyslipidaemia are cIMT and left ventricular morphology, to prevent future development of CVD [9] (Table 1).

**Table 1:** Recommendations for screening dyslipidaemia [11, 12]

Children older than 2 years + 1 or more of the followings:
1. First-grade relatives (men < 55 years and women < 65 years) with previous MACEs;
2. Parents with hypercholesterolemia (Total cholesterol ˃ 240 mg/dL);
3. Parental/grandparental family history not known + 2 or more other risk factors for CAD (including hypertension, cigarette smoking, low HDL cholesterol, obesity, physical inactivity, and diabetes mellitus)

*Abbreviations*: *MACEs* Major Adverse Cardiac Events, including acute coronary syndrome, coronary artery bypass graft or percutaneous coronary intervention, stroke, or sudden cardiac death, *CAD* Coronary Artery Disease

The American Academy of Pediatrics (AAP) recommends to dose serum levels of cholesterol (total, LDL-C and HDL-C) and triglycerides in all children at the age of 10 and to repeat it again at the age of 19 years. In this way it is possible to diagnose hypercholesterolemia and to start early targeted treatment. In the early stages of the disease, the first line of treatment is based on behavioural intervention, through modification of lifestyles and eating habits; in the more advanced stages, with higher levels of serum cholesterol and if the non-pharmacological treatments were not effective, it is possible to start pharmacological treatment with statins [6, 13].

Randomized studies have demonstrated the safety of statins in children and adolescents with FH. Treatment with low doses of statins is therefore indicated in children with the heterozygous form of FH from the age of 8 to 10 years, because it has shown a long-term benefit, with a lower incidence of atherosclerotic CVD in a 40-year-lasting follow-up [1, 14].

## Hypertension

Hypertension in children is a leading determinant of CVD in adults [1]. The estimated prevalence of arterial hypertension in children and adolescents aged 0 to 18 years ranges from 3 to 5% [15] and increases with age accounting for 10–11% in 18-year-old adolescents, a rate which is similar to that of young adults [16]. The incidence of hypertension in adolescents with prehypertension before screening is estimated of 1.4% per year, the same rate as in adults with normal blood pressure (BP) [17, 18] (Table 2).

**Table 2:** Definitions of elevated blood pressure and hypertension in children and adults [19, 20]

**Adults and children ≥ 13 years of age**	
**Elevated blood pressure**	Systolic BP ≥ 120 and diastolic BP ˂ 80 mm Hg
**Stage I hypertension**	Systolic BP ≥ 130 and diastolic BP ≥ 80 mm Hg
**Stage II hypertension**	Systolic BP ≥ 140 and diastolic BP ≥ 90 mm Hg
**Children ˂ 13 years of age**	
**Elevated blood pressure**	Systolic and/ or diastolic BP ≥ 90^th^ percentile for age, sex and height
**Stage I hypertension**	Systolic and/ or diastolic BP ≥ 95^th^ percentile for age, sex and height
**Stage II hypertension**	Systolic and/ or diastolic BP ≥ 95^th^ percentile + 12 mm Hg

*Abbreviations:* *BP* Blood Pressure

**Fig. 1:**
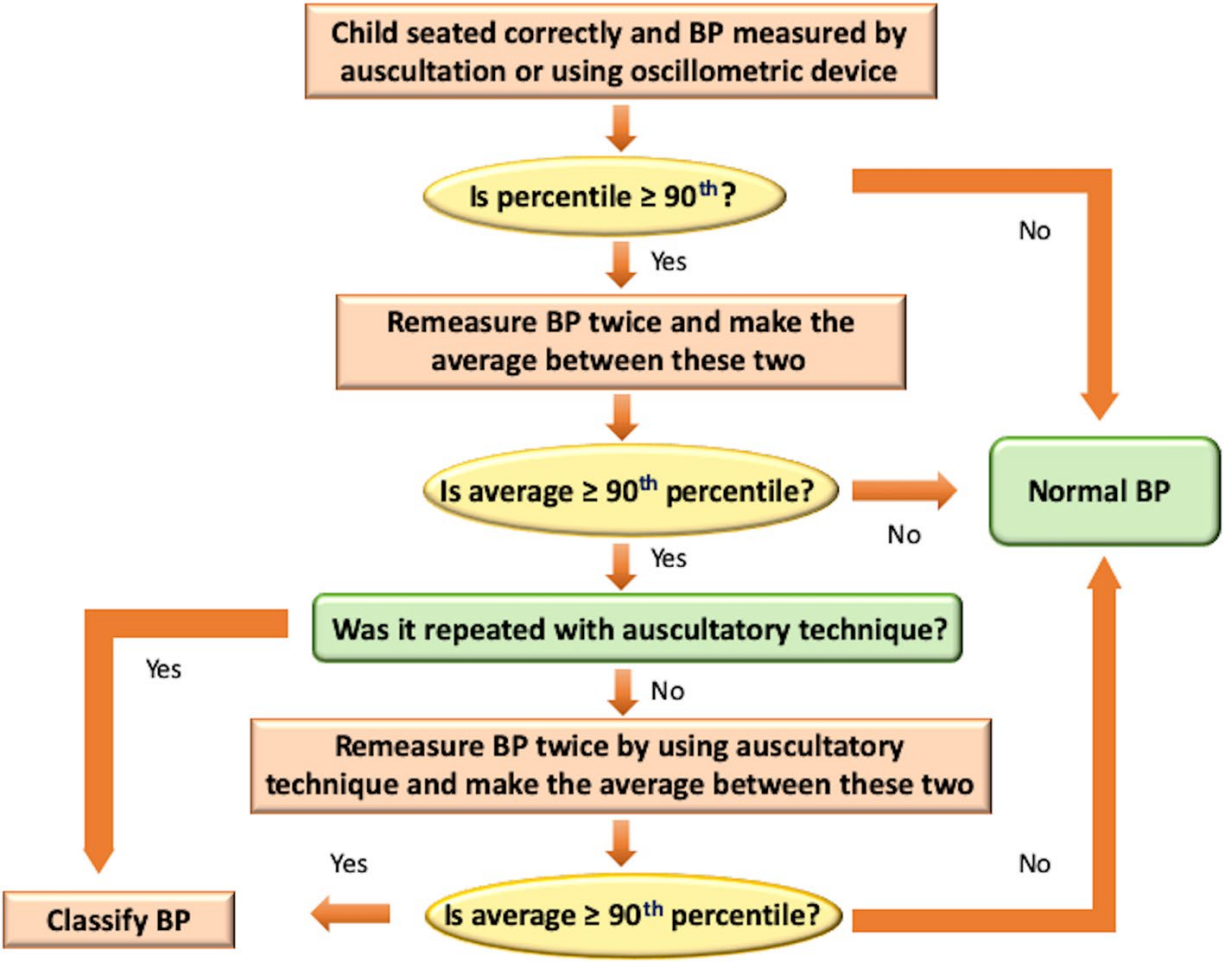
BP measurement algorithm. Adapted from Flynn et al.^22^ Abbreviations: BP, blood pressure

Screening for elevated BP or hypertension (HTN) should begin when the child is 3 years old and diagnosis is confirmed if BP is elevated over 3 visits (Fig. 1) [1].

In children and adolescents aged 6 years or older with a positive family history of HTN, overweight or obese, and/or without history of physical examination findings suggestive of a secondary hypertension, we should diagnose primary HTN avoiding an extensive evaluation for secondary causes [1].

Renal and reno-vascular diseases are the most common causes of secondary HTN in younger children. Among cardiac causes, aortic coarctation is usually associated with HTN and right arm BP that is 20 mm Hg (or more) greater than the lower extremity BP. Other causes of secondary HTN are endocrinological, environmental, associated with neurofibromatosis or medication related [19].

At the present time, no prospective controlled studies are available supporting that HTN treatment in children reduces cardiovascular risk in adulthood. However, promising evidence derives from some prospective controlled studies in children. It has been demonstrated that in youth with elevated BP, reducing their BP to normal adult values leads to cIMT measurements in adulthood, like adults with persistently normal BP [21].

One year of antihypertensive treatment with angiotensin converting enzyme inhibitors (ACEi) and hydrochlorothiazide was shown to significantly decrease BP, left ventricular mass index, prevalence of LVH and microalbuminuria [22].

In children with hypertension, a 1-year antihypertensive treatment with lifestyle intervention and ACEi or angiotensin II receptor blocker (ARB) resulted in a significant decrease of BP, LVH and cIMT [23].

According to the AAP, the first approach to hypertension is a 6-month lifestyle intervention. In case of failure of this approach or symptomatic hypertension, or any stage of hypertension associated with type 1 diabetes mellitus (DM) or chronic kidney disease (CKD), anti-hypertensive treatment should be started with a single medication among ACEi, ARB, long-acting calcium channel blocker, or a thiazide diuretic. If it is not possible to reach the BP target of < 90^th^ percentile or < 130/80 mmHg with the maximal dose of the first agent, if the patient is 13 years old or older, a second medication can be associated and usually a thiazide diuretic is indicated. An ACEi or ARB usually is the most appropriated first-line antihypertensive medication in children affected by CKD, proteinuria, or DM [1].

Echocardiography is recommended to exclude LVH when considering pharmacologic therapy [1].

## Congenital heart diseases

Some studies report that people with structural and functional abnormalities of heart are at greater risk of developing atherosclerosis and all types of CVD, including heart failure, myocardial infarction, stroke, transient ischemic attacks, aortic aneurysms, and peripheral vascular diseases in adulthood.

Congenital heart defects more frequently associated with premature cardiovascular risk in adulthood than the general population are obstructive lesions of the left ventricle and aorta, cyanotic congenital heart defects, including Eisenmenger syndrome, and coronary artery anomalies [24]. Moreover, the presence of significant aortic stenosis can be accompanied by left ventricular hypertrophy, which is a known independent cardiovascular risk factor in adulthood [1, 25].

In addition, in patients with non-severe congenital heart diseases (CHD) who survive into adulthood, acquired heart disease, particularly ischemic heart disease, represent the most common cause of death. Instead, the most common causes of death in patients with severe congenital heart disease are the underlying heart diseases and any acquired CVD, which lead to lower life expectancy than general population. Therefore, there are differences based on severity of the underlying congenital lesion. Furthermore, hypertension and hyperlipidaemia are more present in patients with non-serious CHD [26].

It has been also shown that patients with cyanogenic congenital heart disease have plaque-free coronary arteries and lower cIMT measurements [1].

All these evidences support the low risk of developing atherosclerosis in adulthood of these patients. Thus, it is important, especially in patients with non-severe CHD, to monitor risk factors for CVD with close long-term follow-up [26].

Several studies showed that in adult patients with cyanogenic congenital heart disease, the lipid profile, the cardiovascular risk score and the prevalence of subclinical carotid and coronary atherosclerosis were like the general population. Previous studies suggested that patients with CHD have a lower risk of atherosclerosis, probably due to hypoxemia, which leads to an increase in the production of anti-atherogenic factors like nitric oxide, hyperbilirubinemia, and thrombocytopenia [27].

## Congenital coronary anomalies

Congenital coronary anomalies, isolated or in association with other congenital heart defects, could lead to the development of early atherosclerosis [28]. The importance of primary prevention (diet control, aerobic physical exercise, and smoking cessation) is well known to be effective in reducing cardiovascular risk in adulthood, however, also the screening and treatment of hypertension, DM and hyperlipidemia play an important role [1].

## Kawasaki disease

Kawasaki disease (KD) is an acute systemic vasculitis, which is the most frequent cause of acquired paediatric heart disease in industrialized countries. It affects coronary arteries, leading to the formation of coronary aneurysms in severe cases. The incidence has increased in recent years (up to 30 per 100,000 children in the United States) [29, 30].

Aetiology is unknown. Coronary artery aneurysm (CAA) occurs in 20–25% of untreated children. These patients present a risk of developing cardiovascular pathologies, but it is not clear whether the accelerated atherosclerosis process can be related to subsequent coronary complications [1, 31].

Individuals who develop CAA have an increased cIMT, endothelial dysfunction and an increase in arterial stiffness, although there are discordant data in the literature, particularly regarding low-risk groups [32, 33].

Ten out of 13 studies on KD have shown that endothelial dysfunction develops in some patients with KD (Table 3). In fact, these studies showed a lower incidence of fibromuscular dysplasia in affected patients compared to controls, regardless of ethnicity, time elapsed from the acute clinical presentation of the disease and the presence of coronary aneurysms [29].

**Table 3:** Methods for studying subclinical vascular changes in Kawasaki disease and their association with cardiovascular risk [29]

	**Abbreviations**	**Structure or function measured**	**Increased cardiovascular risk**
**Carotid intima-media thickness**	cIMT	Intima-media complex of the carotid artery	Increased carotid intima-media thickness
**Flow-mediated dilatation**	FMD	Endothelial function	Smaller percentage increase in the diameter of the brachial artery following cuff release after suprasystolic occlusion
**Peripheral arterial tonometry**	PAT	Endothelial function	Smaller pulse volume increase in the digital arteries following cuff release after suprasystolic brachial artery occlusion
**Pulse wave velocity**	PWV	Arterial stiffness	Increased pulse wave velocity

Atherosclerosis and KD have chronic inflammation of the arterial wall in common, while they differ in lipid deposition in the arterial intimate area, which is not usually found in KD [34].

Indeed, as shown by a recent cardiac magnetic resonance study, used for the analysis of the concentration of lipoprotein particles, subjects with KD do not have a worse lipid profile than the general population. In fact, it seems that KD can cause an acute inflammatory lesion which then leads to the development of atherosclerosis as a "response to the lesion” [35]. It is important to advise individuals with previous KD to maintain a healthy lifestyle and minimize modifiable cardiovascular risk factors such as smoking, hyperlipidaemia, hypertension, hyperglycaemia, and obesity [29].

## Chronic kidney disease

About 10,000 children are treated with dialysis in the United States and about 1,000 children receive a kidney transplant every year [36]. Although survival of subjects with an early onset of CKD has improved over the last decades [37], mortality rates still amount of 30 times in children treated with long-term dialysis and 10 times in young kidney transplant recipients if compared with general paediatric population [1].

CVD is the leading cause of death in children with end-stage renal disease [1]. In fact, in these patients several risk factors contribute to the development of early damages in blood vessels and in cardiac function [38]. Hypertension is the most important risk factor for CVD during early CKD, however, during dialysis, fluid overload, mineral bone disease, and anaemia play the main role [1].

Arrhythmias, valvular heart disease, cardiomyopathy and sudden cardiac death have been observed in children with stage 5 CKD [39].

**Fig. 2:**
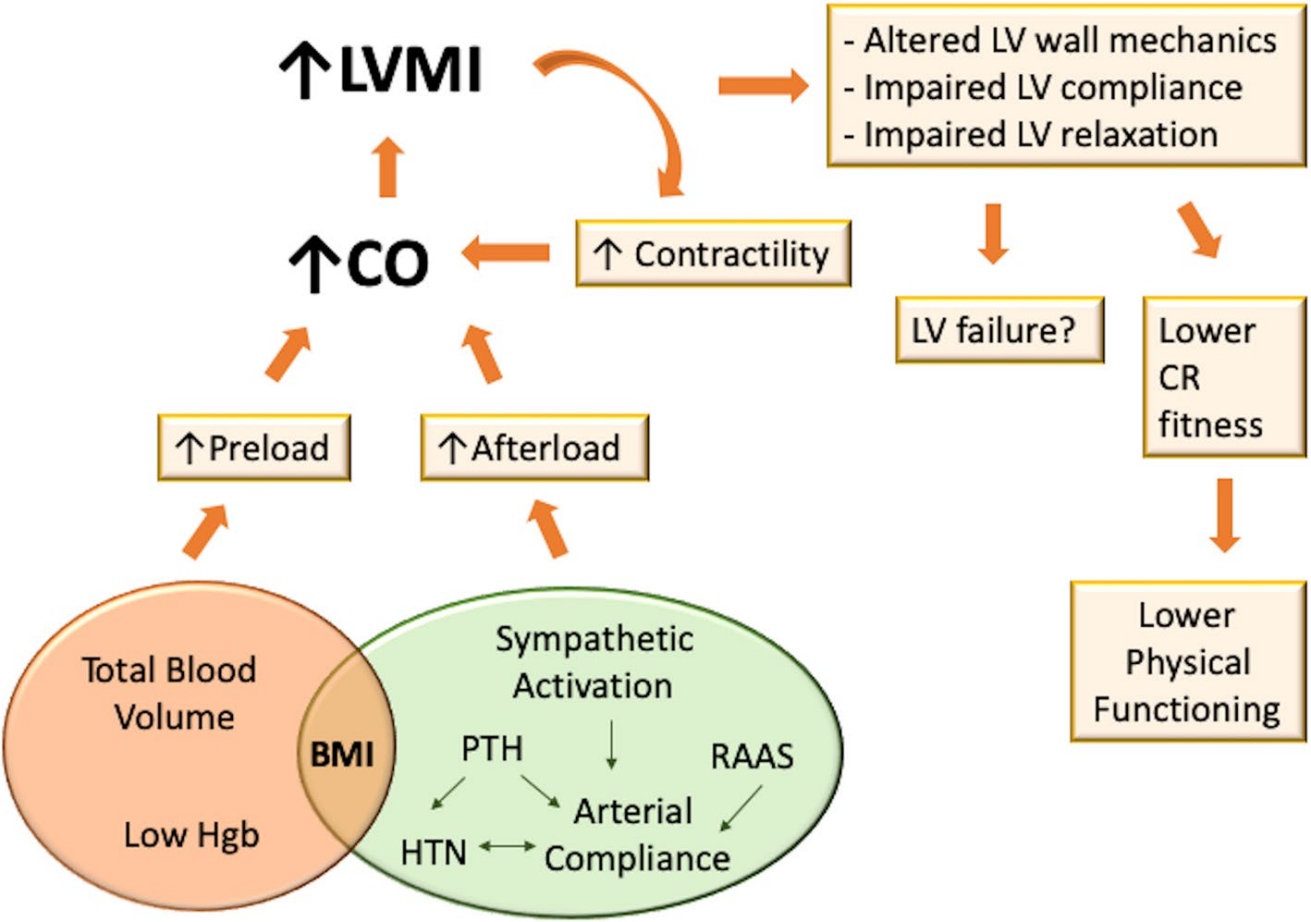
Cardiac adaptation in paediatric chronic kidney disease. Left ventricular adapts through concentric and eccentric geometry patterns in response to increasing afterload and preload. Afterload increases because of a higher systemic arterial resistance, due to hypertension, and a reduced large-vessel compliance, resulting in an increase of the left ventricular wall thickness and therefore in a concentric left ventricular hypertrophy. Preload increases because of intravascular volume expansion, due to volume overload, anaemia and even the presence of an arteriovenous fistula, leading to eccentric hypertrophy. In addition, oxidative stress, enhanced renin–angiotensin–aldosterone system activation, and chronic inflammation have been shown to be responsible for LVH in murine models. Abbreviations: BMI, body mass index; CO, cardiac output; CR, cardiorespiratory; Hgb, haemoglobin; HTN, hypertension; LV, left ventricular; LVMI, left ventricular mass index; PTH, parathyroid hormone; RAAS, renin-aldosterone-angiotensin system

Several alterations in cardiovascular structure and function occur in the early stages of CKD, most likely because of an attempt to adapt to the hemodynamic and biochemical anomalies typical of CKD (Fig. 2) [40–42].

Recent studies also have suggested that Fibroblast growth factor 23 may contribute to LVH directly inducing myocyte hypertrophy [43] and correlates with decreasing glomerular filtration rate [44].

Young women with CKD are at greater risk for mortality than young men [45]. A study conducted on 681 children with CKD revealed that indexing left ventricular mass to height showed a higher proportion of LVH among girls, while estimates of LVH based on estimated lean body mass showed no sex differences [46].

Kidney Disease Improving Global Outcomes provides recommendations for the management of the most common CVD risk factors in children with CKD.

A BP-lowering treatment is recommended, especially in children with proteinuria, in order to consistently reach systolic and diastolic readings less than or equal to the 50^th^ percentile for age, sex, and height, unless achieving these targets leads to signs or symptoms of hypotension. In these patients, ARB or ACEi should be used, irrespective of the level of proteinuria [46].

In children with newly identified CKD, an assessment of lipid profile is recommended. In all children with CKD, an annual evaluation of fasting lipid levels is indicated. However, statins or statin/ezetimibe combination should not be initiated before 18 years of age.

In children with CKD and hypertriglyceridemia, therapeutic lifestyle changes are recommended [38].

The most dangerous condition for the development or worsening of CVD is dialysis, therefore, an early transplantation is recommended to avoid long-term consequences of dialysis. However, transplantation does not represent a definitive solution because cardiovascular risk persists after kidney transplantation [38]. In case of necessity of a long-term dialysis, a careful follow-up and management of hypertension, dyslipidemia, abnormal mineral metabolism, inflammation and other dialysis complications is a must [1].

## Childhood cancer

Thanks to the increasingly available innovative treatments, the rate of adults survived to cancer in childhood is improving [47], however, the late morbidity and mortality rates are still elevated [48]. Childhood cancer survivors (CCS) have a risk of dying of CVD 8 to 10 times higher than age-matched control subjects [49]. The Childhood Cancer Survivor Study revealed that CCS had a tenfold greater risk of coronary artery disease (CAD), a ninefold greater risk of cerebrovascular accident and a 15-fold greater risk of congestive heart failure in comparison with their siblings [50].

CCS have higher fat mass, lower lean body mass, greater insulin resistance, lower carotid distensibility and compliance, and increased arterial stiffness than their siblings [50]. The pathogenesis of the increased cardiovascular risk in these subjects is multifactorial and previous anti-tumoral treatments play an important role in it. For instance, in patients who received a 1550 centigray total body or chest or abdominal radiotherapy the risk of developing congestive heart failure, myocardial infarction, pericardial disease and valvular abnormalities is 2–sixfold greater than in CCS not exposed to radiation [51]. Hematopoietic stem cell transplantation leads to a 2 to 3 times greater risk of dying of CVD mortality and increased probability of developing cardiomyopathy, congestive heart failure, cerebrovascular accident, CAD and rhythm disorders [52, 53].

Brain tumours and respective treatments, such as radiotherapy or brain surgery, can damage the hypothalamus and pituitary gland, resulting in several endocrine disorders, mostly growth hormone deficiency, which is well known to enhance the components of the metabolic syndrome [54–56].

Conformal radiation therapy (CRT) > 30 Gy causes long-term central hypogonadism in 20–30% and central hypothyroidism in 3–9% of CCS [54, 55]. Thyroid malignancies or neck and mantle radiation for other cancer types cause primary hypothyroidism [57], leading to increased adiposity, hypertension, due to an increase in peripheral vascular resistance, and dyslipidemia [58].

Adrenal insufficiency occurs in 3–6% of patients treated with > 30 Gy CRT [54] and may be also caused by high-dose steroid treatment [55].

It should also be considered that some chemotherapy medications, such as ifosfamide and methotrexate, are nephrotoxic and therefore renal insufficiency and hypertension are other frequent comorbidities [1].

To date, several guidelines for the management of cardiovascular risk in CCS are available, however their weakness is the lack of large prospective studies. Nevertheless, some concepts are very useful to orientate physicians in their clinical practice.

The Children’s Oncology Group recommends screening CCS patients with a fasting lipid profile and fasting glucose or glycated haemoglobin every 2 years [59].

Lifestyle intervention for the maintenance of appropriate weight, heart-healthy diet, adequate physical activity, and avoidance of smoke exposure should be strongly encouraged and treatment of cardiovascular risk factors should be personalised according to patients’ previous cancer therapies [59].

## Conclusions

Cardiovascular risk affects all phases of life and its prevention and screening should be started in early childhood. Dyslipidaemia, hypertension, congenital heart diseases, congenital or acquired coronary anomalies, CKD and childhood cancer survival represent only a small group of cardiovascular risk factors. Therefore, a lifelong, multidisciplinary, and personalized approach is indicated to guarantee the best perspectives for children’s future.

## Data Availability

Not applicable.

## Abbreviations

CVD: Cardiovascular diseases
cIMT: Carotid intima-media thickness
LDL-C: LDL cholesterol
FH: Familial hypercholesterolemia
LVH: Left ventricular hypertrophy
MACEs: Major adverse cardiac events
CAD: Coronary artery disease
AAP: American Academy of Pediatrics
BP: Blood pressure
HTN: Hypertension
ACEi: Angiotensin converting enzyme inhibitors
ARB: Angiotensin II receptor blocker
DM: Diabetes mellitus
CKD: Chronic kidney disease
CHD: Congenital heart diseases
KD: Kawasaki disease
CAA: Coronary artery aneurysm
CCS: Childhood cancer survivors
CRT: Conformal radiation therapy
